# Prediction model for chemical explosion consequences via multimodal feature fusion

**DOI:** 10.1186/s13321-025-01060-x

**Published:** 2025-08-05

**Authors:** Yilin Wang, Beibei Wang, Yichen Zhang, Jiquan Zhang, Yijie Song, Shuang-Hua Yang

**Affiliations:** 1 College of Jilin Emergency Management, Changchun Institute of Technology, Changchun, 130012 China; 2 School of Environment, Northeast Normal University, Changchun, 130117 China; 3 Department of Computer Science, University of Reading, Reading, RG6 6AH UK

**Keywords:** Chemical explosion accidents, Molecular structure, SMILES, Multimodal feature fusion, Machine learning

## Abstract

**Abstract:**

Chemical explosion accidents represent a significant threat to both human safety and environmental integrity. The accurate prediction of such incidents plays a pivotal role in risk mitigation and safety enhancement within the chemical industry. This study proposes an innovative Bayes-Transformer-SVM model based on multimodal feature fusion, integrating Quantitative Structure–Property Relationship (QSPR) and Quantitative Property-Consequence Relationship (QPCR) principles. The model utilizes molecular descriptors derived from the Simplified Molecular Input Line Entry System (SMILES) and Gaussian16 software, combined with leakage condition parameters, as input features to investigate the quantitative relationship between these factors and explosion consequences. A comprehensive validation and evaluation of the constructed model were performed. Results demonstrate that the optimized Bayes-Transformer-SVM model achieves superior performance, with test set metrics reaching an R^2^ of 0.9475 and RMSE of 0.1139, outperforming alternative prediction models. The developed model offers a novel and effective approach for assessing explosion risks associated with both existing and newly developed chemical substances. The model enables rapid explosion consequence assessment for chemical storage or transport scenarios, supporting safety-by-design frameworks.

**Scientific Contributions:**

This study constructed a Bayes-Transformer-SVM model for predicting the consequences of hazardous chemical explosions. The model utilized SMILES encoding and Gaussian16 quantum chemical descriptors, combined with leakage condition scenario parameters, achieving excellent performance. Its core lies in the establishment of a multimodal fusion theoretical framework, breaking through the limitations oftraditional cross-modal correlation analysis; the development of an optimized architecture that combines Transformer feature extraction and SVM regression; highlighting the potential application of the model in chemoinformatics; and enabling the prospective assessment of the explosion risks of unknown chemicals, supporting a safety-oriented design concept.

**Supplementary Information:**

The online version contains supplementary material available at 10.1186/s13321-025-01060-x.

## Introduction

An explosive chemical is a solid or liquid substance capable of undergoing a chemical reaction that rapidly generates gas with sufficient temperature, pressure, and speed to damage its surroundings [1]. Specific dangers associated with their production and processing processes can result in significant accidents and losses [2]. Among chemical process safety hazards, explosion events are prioritized as the highest-risk phenomena in industrial settings, representing over 60% of major accidents and inducing cascading failures through blast waves and fragment projection [3, 4]. Concurrently, economic advancement gives rise to a multitude of environmental safety concerns, with incidents occurring regularity due to a combination of human-made, managerial, and objective factors [5]. Consequently, a comprehensive examination of the risk of combustion and explosion by hazardous chemicals is imperative for enhancing chemical site safety and reducing the probability of accidents.

To prevent and control potential accident consequences involving explosive chemicals during initial stages, timely and accurate prediction of their diffusion range is crucial for emergency planning and consequence analysis. Accident outcomes can be forecasted using empirical models, computational fluid dynamics (CFD), or integrated models. While empirical models enable rapid prediction, their accuracy is limited as they neglect the impact of surface roughness—a critical factor influencing the evaporation rate of large liquid spills. CFD simulations overcome this limitation by capturing surface roughness effects and enabling the study of obstacles through geometrically accurate representations. However, configuring geometric and boundary conditions is highly time-consuming, and the computations are resource-intensive, making CFD less suitable for immediate emergency response predictions. 

PHAST is one of the most popular process hazard analysis software, and the integrated model it uses provides a trade-off between accuracy and computational cost. Its dispersion prediction tool utilizes the UDM (Unified Dispersion Model) to simulate the full process from initial release to far-field dispersion. The software also models rainfall and subsequent evaporation. Experimental validations of the PHAST UDM module demonstrate high prediction accuracy, which has been widely substantiated [6, 7]. Many researchers have also used the data generated by the PHAST software to predict the diffusion of flammable chemicals and achieved very good results [8, 9].

At present, consequence prediction requires an appropriate framework. A highly linear relationship between input parameters and consequence goals is more conducive to the development of prediction models. Quantitative structure–property relationship (QSPR) techniques were the extensively utilized theoretical approaches for predicting properties associated with fire and explosion [10, 11], which can elucidate the mathematical relationships between structural attributes and properties at the quantum chemical level and exhibited superior accuracy and reliability compared to alternative predictive approaches [12]. A significant number of scholars have employed QSPR to predict the hazardous properties of chemicals, including auto-ignition temperature [13, 14], minimum ignition energy [15, 16], flammability limit [17, 18], heat of combustion [19, 20], and flash point [21, 22]. Nevertheless, the application of QSPR models at the macroscopic level is constrained by certain limitations [23].

The Quantitative Property-Consequence Relationship (QPCR) approach, introduced by Jiao and colleagues in 2020, represents another promising approach for developing accident consequence prediction models, which determines the consequences of accidents by examining the properties of substances [24]. The method employs situational and chemical properties as property descriptors for predictive modeling and has been applied on numerous occasions in the prediction of fires and toxic dispersion [9, 25].

However, due to the insufficient characterization of hazard properties for complex chemicals in chemical explosions, achieving accurate predictions using either method alone is challenging. Therefore, this study integrates the QSPR and QPCR methods to establish an integrated model based on multimodal feature fusion. This model aims to predict chemical explosion accident consequences, offering an improved approach for consequence analysis, risk assessment, and the development of emergency response strategies.

Selecting the appropriate algorithm is the basis for developing multimodal feature fusion prediction models [26]. Machine learning has been proven to have higher accuracy and efficiency [9, 27]. Pan et al. empirically validated a molecular topology fingerprint-based ensemble learning framework for predicting compound-specific lower flammability limits (LFL) [28] and auto-ignition temperatures (AIT) [29]. Parallel investigations by Wang et al. demonstrated combustion limit prediction through comparative analysis of support vector regression (SVR) and multiple linear regression (MLR) methodologies [17, 30]. These established approaches employ conventional descriptor-driven modeling architectures, implementing multidimensional feature reduction techniques for high-dimensional feature space optimization through recursive feature elimination algorithms.

The progression of computational frameworks has driven transformative approaches in cheminformatics, where researchers have innovated model frameworks via natural language processing (NLP) architectures using SMILES-based molecular encodings. Kim et al. integrated sequence conversion algorithms with variational autoencoder architectures to synthesize novel chemical entities aligned with predefined physicochemical criteria [31]. Aahil Khambhawala et al. developed Transformer-based architectures utilizing SMILES notation descriptors to forecast ionic compound phase-transition temperatures [32]. These methodological adaptations introduced modified transformer architectures to address inherent limitations of SMILES notation in capturing stereochemical complexity. The central research thrust examines how nonlinear attention weights within neural architectures establish interpretable correlations between molecular topology and target macroscopic properties. Models using only basic structural features (like SMILES codes or functional group counts) are easier to use but often less accurate and provide less chemical insight than models including quantum chemical descriptors, which explain the underlying chemistry [33]. Current scientific research either uses molecular descriptors or SMILES strings for predictions, ignoring the collaborative integration of multimodal molecular representations that could potentially leverage complementary fields of cheminformatics knowledge through hybrid architecture innovations.

To systematically investigate and predict the correlation between molecular determinants and consequences in chemical explosion events, this paper constructs a prediction model based on multimodal feature fusion, employing the Bayes-Transformer-SVM (BTS) hybrid method. Bayesian-optimized Transformer encoder for capturing long-range dependencies in SMILES sequences, enhancing feature extraction beyond conventional descriptor-driven models. By integrating molecular descriptors, SMILES encodings, and leakage condition parameters, the BTS model achieves accurate prediction of explosion diameter, overcoming limitations of single-modality representations. Consequently, the model aims to elucidate the relationship between molecular structure and explosion consequences, enhancing interpretability. While this represents a significant improvement over traditional artificial intelligence algorithms in prediction accuracy, the model’s interpretability remains limited.

## Methodology

### Dataset description

Prior to establishing the BTS prediction model, a comprehensive chemical diffusion consequence database accounting for various leakage scenarios involving different explosive chemicals is required. This study employed the PHAST UDM simulation methodology to construct a database of chemical explosion impact ranges (denoted as “explosion diameter”) [27, 34]. The term “explosion diameter” describes the spatial extent of an explosion event, with the target overpressure intensity at a specified distance set at 2068 Pa. Leakage scenario parameters comprise three main components: source conditions (released material, location, quantity, etc.), meteorological conditions (wind speed, atmospheric temperature, humidity, etc.), and leakage conditions (leakage aperture/size), with detailed settings provided in Table 1. By simulating diverse leakage scenarios and varying the four key parameters—leakage aperture, release quantity, temperature, and pressure—108 unique release conditions were generated, while unspecified parameters retained default values. Due to constraints within the software’s database, the dataset was expanded to the maximum extent possible under these technical limitations. Subsequently, 108 leakage scenarios across 40 explosive chemicals were simulated, resulting in a database containing 4016 effective explosion scenario data entries. As model development relied exclusively on this single simulated data source, internal model consistency was ensured, thereby facilitating more accurate predictions, with the explosion diameter result can be found in Table S1 in the support information.

**Table 1:** Parameter specification of explosive chemicals in PHAST simulations

Weather category	1.5/F
Atmospheric temperature ( °C)	20
Surface temperature ( °C)	20
Temperature ( °C)	0、20、50、100
Pressure (psi)	100、500、1000
Leak size (in)	10、25、50
Material quantity (m^3^)	10、20、100

To quantitatively characterize structural diversity, we computed Tanimoto similarity indices (range: 0–0.67) show in Figure S1. Despite limited compound count, the dataset covers 12 major functional groups (alkanes, alkenes, alcohols, aromatics, Amines, Ketones, etc.) with simulated values ranging from 16.473 to 3355.77, ensuring sufficient chemical space coverage.

### Representation of molecular structure

#### Molecular descriptors

Molecular descriptors are computed using Gaussian 16 software. In this study, the task types for each computational task were geometry optimization and frequency and thermochemical analysis, and the method was density flooding (B3LYP) with a basis set of 6-31G (d) [35]. The computed molecular descriptors need to be culled to avoid “chance correlation”, and the selection of important chemical descriptors can improve the performance of model [36]. For molecular descriptors with a high covariance between them, one of them is deleted, and molecular descriptors that have a value of 0 for most compounds are deleted. The resulting molecular descriptors are listed in Supporting Information Table S1.

#### Simplified molecular input line entry system

The simplified molecular input line entry system (SMILES) constitutes a line notation system for unambiguously encoding molecular topology using ASCII characters [37]. SMILES strings encapsulate essential structural characteristics of chemical entities, including atomic connectivity, bond orders, branching patterns, and cyclic/aromatic substructures through syntax-based conventions. Atomic constituents are denoted through standardized atomic symbols, with covalent interactions explicitly represented by hyphen (“-”), equals (“ = ”), and hash (“#”) symbols corresponding to single, double, and triple bonds respectively [31]. Structural isomerism is resolved.

Through parenthetical notation for branching hierarchy. These syntactic conventions enable comprehensive representation of a molecule’s complete topological configuration within a linear string notation format.

### Deep learning algorithms

In this paper, the Bayes-Transformer-SVM method is chosen to predict the results of the explosion.

#### Bayesian optimization algorithm

Hyperparameter tuning is critical for optimizing ML models. Bayesian optimization efficiently selects hyperparameters by globally searching for optimal solutions using an unknown objective function. It requires only input–output definitions, not internal function details. The process updates the objective function’s posterior distribution via sequential sampling:

1 $$p\left(f|{D}_{1:t}\right)=\frac{p\left({D}_{1:t}|f\right)p\left(f\right)}{p\left({D}_{1:t}\right)}$$

where $f$ is the unknown objective function, ${D}_{1:t}=\left({x}_{1},{y}_{1}\right) , \left({x}_{2},{y}_{2}\right), \cdots , \left({x}_{t},{y}_{t}\right)$ is the observed set, where ${x}_{t}$ is the decision vector; ${y}_{t}=f\left({x}_{t}\right)+{\varepsilon }_{t}$ is the observation.

The Bayesian optimization algorithm consists of two basic components: a probabilistic agent model and a collection function [38]. The probabilistic agent model is based on a finite set of observations constantly updating the prior and using Bayes’ theorem to estimate the posterior probability distribution $p\left(f|{D}_{1:t}\right)$, this encompasses additional data details and estimates the distribution pattern of the intended black-box function.

#### Transformer framework

**Fig. 1:**
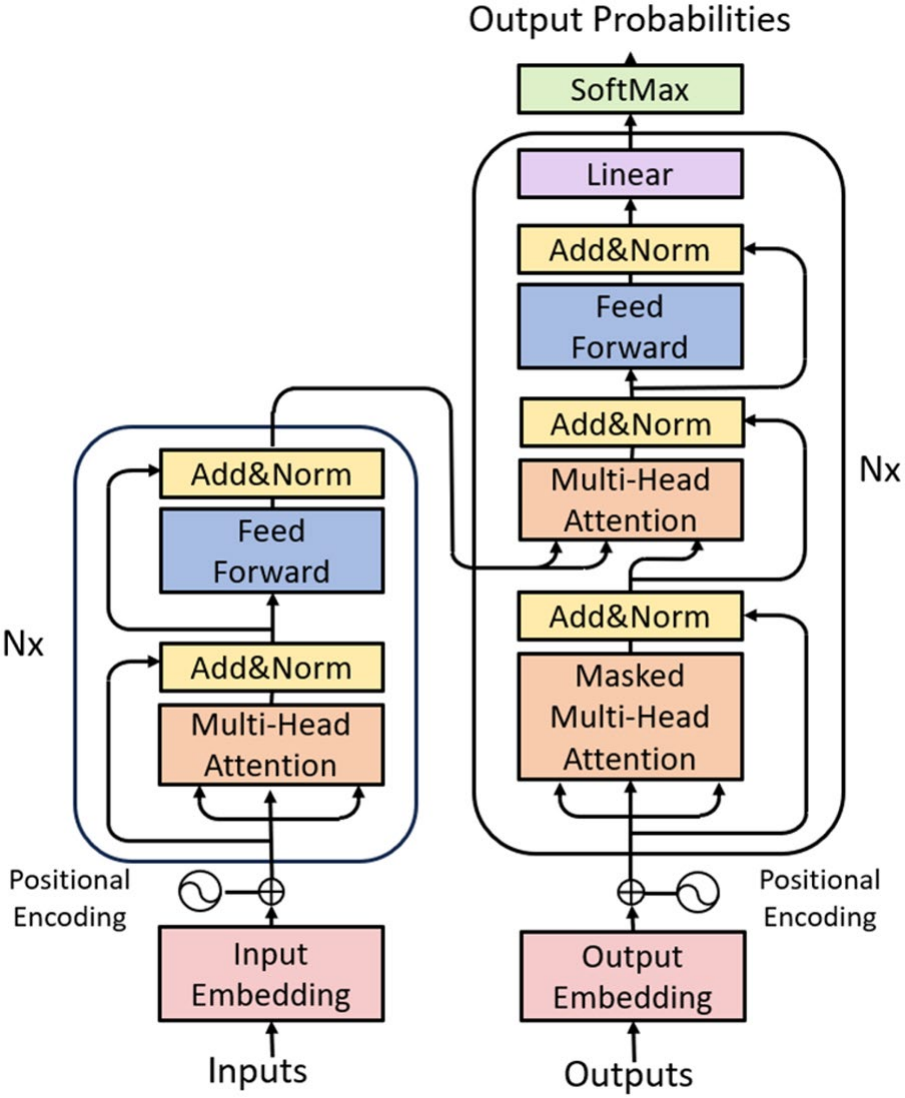
Transformer model structure diagram

The Transformer architecture, first introduced by Google researchers in their seminal 2017 work [39], represents a foundational framework in natural language processing. This model employs a self-attention mechanism to dynamically model interdependencies between input and output sequences, eschewing the sequential processing constraints inherent in recurrent neural networks (RNNs). As depicted in Fig. 1, the canonical Transformer configuration comprises stacked encoder-decoder modules with homogeneous substructures. Each encoder layer integrates multi-head attention and position-wise feed-forward networks, whereas the decoder incorporates an additional masked attention mechanism to preserve autoregressive property during inference. Researchers have extended this framework for SMILES sequences [40] and multimodal inputs via dual-encoder designs with parametric attention fusion [41].

Attention mechanisms, initially explored in computer vision [42], were formalized in Transformers through multi-head self-attention [43]. This processes inputs in parallel across representation subspaces using learnable matrices: Query (*Q*), Key (*K*), and Value (*V*). The scaled dot-product attention is computed as:

2 $$Attention\left(Q,K,V\right)=softmax\left(\frac{Q{K}^{T}}{\sqrt{{d}_{k}}}\right)\bullet V$$

Multi-head attention concatenates outputs from multiple such attention heads (head_i_), linearly projected via weight matrices.

#### Support vector machine model (SVM)

Support Vector Machines (SVM), introduced by Vapnik et al. [44], are discriminative models based on VC theory and structural risk minimization [45]. Originally for linear binary classification, SVM finds the optimal separating hyperplane maximizing the margin between classes. For nonlinear problems, SVM employs kernel functions to map data into a higher-dimensional feature space where this linear separation is possible.

For regression (Support Vector Regression—SVR), the goal is to find a hyperplane minimizing the deviation of all data points within a tolerance ε. The regression function is

3 $$f\left(x\right)=\sum_{i=1}^{l}{w}_{i}\cdot {\phi }_{i}\left(x\right)+b$$

where *ϕ*_*i*_*(x)* are nonlinear transformations, and *w*_*i*_, *b* are parameters. Parameters are determined by minimizing the structural risk:

4 $$R\left(C\right)=C\frac{1}{N}\sum_{i=1}^{N}{L}_{\varepsilon }\left({d}_{i},{y}_{i}\right)+\frac{1}{2}{\Vert w\Vert }^{2}$$

5 $${L}_{\varepsilon }\left({d}_{i},{y}_{i}\right)=\left\{\begin{array}{c}\left|d-y\right|-\varepsilon \left(\left|d-y\right|\ge \varepsilon \right)\\ 0 \left({\text{others}}\right)\end{array}\right.$$

In Eq. (3), $C\frac{1}{N}\sum_{i=1}^{N}{L}_{\varepsilon }\left({d}_{i},{y}_{i}\right)$ is the empirical error, measured by the *ε-*insensitive loss function ${L}_{\varepsilon }\left({d}_{i},{y}_{i}\right)$. $\frac{1}{2}{\Vert w\Vert }^{2}$ is used to measure the flatness of the function. c is a regularization constant that determines the trade-off between the training error and the model flatness. When slack variables $\xi$ and ${\xi }^{*}$ are introduced, Eq. (5) can be written as.

6 $$\begin{array}{c}Max R\left(w,{\xi }^{*}\right)=\frac{1}{2}{\Vert w\Vert }^{2}+C\sum_{i=1}^{n}\left(\xi ,{\xi }^{*}\right) \\ \left({y}_{i}-wx-b\le \varepsilon +{\xi }_{i},wx+b-{y}_{i}\le \varepsilon +{\xi }_{i},{\xi }_{i},{\xi }_{i}^{*}\ge 0\right)\end{array}$$

The decision function is expressed in dual space through kernel substitution:

7 $$\begin{array}{c}f\left(x,{\alpha }_{i}{,\alpha }_{i}^{*}\right)K\left(x{,x}_{i}\right)+b\end{array}$$

8 $$\begin{array}{c}K\left(x{,x}_{i}\right)=\phi \left(x\right)\bullet \phi \left({x}_{i}\right)\end{array}$$

where $K\left(x{,x}_{i}\right)$ denotes a Mercer kernel operator satisfying.

### Combined model prediction

This study proposes for the first time a Bayes-Transformer-SVM hybrid model under a Bayesian optimization framework for joint multimodal data fusion and explosion risk prediction. The model achieves deep fusion of chemical structure features and engineering parameters through a hierarchical architecture: Bayesian optimization first tunes model parameters; an improved Transformer encoder then captures long-range molecular topology dependencies using multi-head self-attention to weight atomic bonding interactions, while engineering parameters like temperature and pressure undergo normalization and feature splicing for dimension alignment with chemical features. Finally, Support Vector Regression (SVR) replaces traditional fully-connected layers to construct the optimal separation hyperplane in high-dimensional feature space. This design mitigates SVM’s local optimality while enabling interpretability through attention mechanisms – a capability absents in standard deep learning models.

### Model validation and evaluation

#### Evaluation of model fitting ability

Four performance metrics were used to evaluate the BST model and baseline models: (1) the cross-validated predictive square correlation coefficient (${Q}_{CV}^{2}$), (2) the coefficient of determination (R^2^), (3) root mean square error (RMSE), and (4) mean absolute error (MAE). The formulas for these metrics are defined as follows:

9 $$\begin{array}{c}{Q}_{CV}^{2}=1-\frac{\sum_{i=1}^{n}{\left(\widehat{{y}_{0}}-\widehat{{y}_{i}}\right)}^{2}}{\sum_{i=1}^{n}{\left(\widehat{{y}_{0}}-\widehat{y}\right)}^{2}}\end{array}$$

10 $$\begin{array}{c}{R}^{2}=\frac{\sum_{i=1}^{n}{\left({y}_{0}-\overline{y }\right)}^{2}-\sum_{i=1}^{n}{\left({y}_{0}-{y}_{i}\right)}^{2}}{\sum_{i=1}^{n}{\left({y}_{0}-\overline{y }\right)}^{2}}\end{array}$$

11 $$RMSE = \sqrt {\frac{{\mathop \sum \nolimits_{i = 1}^{n} \left( {y_{i} - y_{0} } \right)^{2} }}{n}}$$

12 $$MAE = \frac{{\mathop \sum \nolimits_{i = 1}^{n} \left| {y_{i} - y_{0} } \right|}}{n}$$

where ${y}_{i}$ is the simulated value, ${y}_{0}$ is the predicted value and *n* is the number of chemicals in the dataset. $\widehat{{y}_{0}}$ is the predicted value of training set.

#### Model validation standards

In the absence of a validation standard, reference is made to the validation standard of QSPR study. The details are as follows [46, 47]:

Training set: *Q*^*2*^_*LOO*_ > 0.5.

Test set: (1) ${\text{R}}^{2}$ > 0.6; (2) $\left({\text{R}}^{2}-{\text{R}}_{0}^{2}\right)/{\text{R}}^{2} < 0.1$ and 0.85 ≤ k ≤ 1.15 or $(R^{2} - R_{0}^{^{\prime}2} )/R^{2} < 0.1$ and 0.85 ≤ k' ≤ 1.15; (3) $\left| {{\text{R}}_{0}^{2} - {\text{R}}_{0}^{^{\prime}2} } \right| < { }0.3$.

Where *R*^*2*^ is the coefficient of determination of the test set; ${\text{R}}_{0}^{2}$ is the squared correlation coefficient for the zero-intercept linear regression between predicted and experimental values; *k* is lope parameter of the constrained linear regression through the origin in the test set; ${\text{R}}_{0}^{^{\prime}2}$ is the complex correlation coefficient of experimental and predicted values over the origin in the test set. Complex correlation coefficient of linear regression over origin.

### Model application domain

Prior to deployment, defining the model’s application domain is critical as even high-performing models may not cover all chemical compounds. This study employs leverage values [48] visualized via Williams plots [49] – with standardized residuals on the vertical axis and leverage values (${h}_{i}$) on the horizontal axis – to delineate applicability. The values of ${h}_{i}$ and h* are calculated through the application of the following formulas:

13 $$\begin{array}{*{20}c} {h_{i} = X_{i} \left( {X^{T} X} \right)^{ - 1} X_{i}^{T} (i = 1,2,3, \ldots ,n)} \\ \end{array}$$

In this context, *Xi* represents the row vector from the molecular descriptor matrix corresponding to compound *i*, whereas *X* denotes the molecular descriptor matrix for the training set, which contains a variety of compounds. Furthermore, n represents the total number of sample sets*.*

14 $$\large \large \begin{array}{c}{h}^{*}=\frac{{3}\text{(k+}{1}\text{)}}{m}\end{array}$$

where k represents the count of molecular descriptors; m denotes the number of samples in the training dataset.

### Mechanistic explanation of the model

Understanding the correlation between the molecular structure of compounds and their accident consequences, explaining the meaning of the model weights and providing new ideas for the study of compound related reaction mechanisms are the main tasks of the mechanistic interpretation of the model. To clarify the degree of influence and the magnitude of the contribution value of each molecular descriptor on the explosion diameter results, and to grasp the pattern of its influence on the explosion results. In this study, Shapley Additive Explanations (SHAP) [50] was used to explain the degree of influence of each molecular descriptor in the model. It provides an intuitive way of interpreting the predictions of machine learning models and helps the user to understand the degree of dependence of the model on different features. Its main principle is based on the local interpretability model, which quantifies the contribution of features to the final prediction results by evaluating all possible combinations of the feature space, calculating the Shapley value of each feature, and weighting the Shapley value according to the different values of the features to derive the average contribution.

## Results and discussion

### Data analysis and preprocessing

**Fig. 2:**
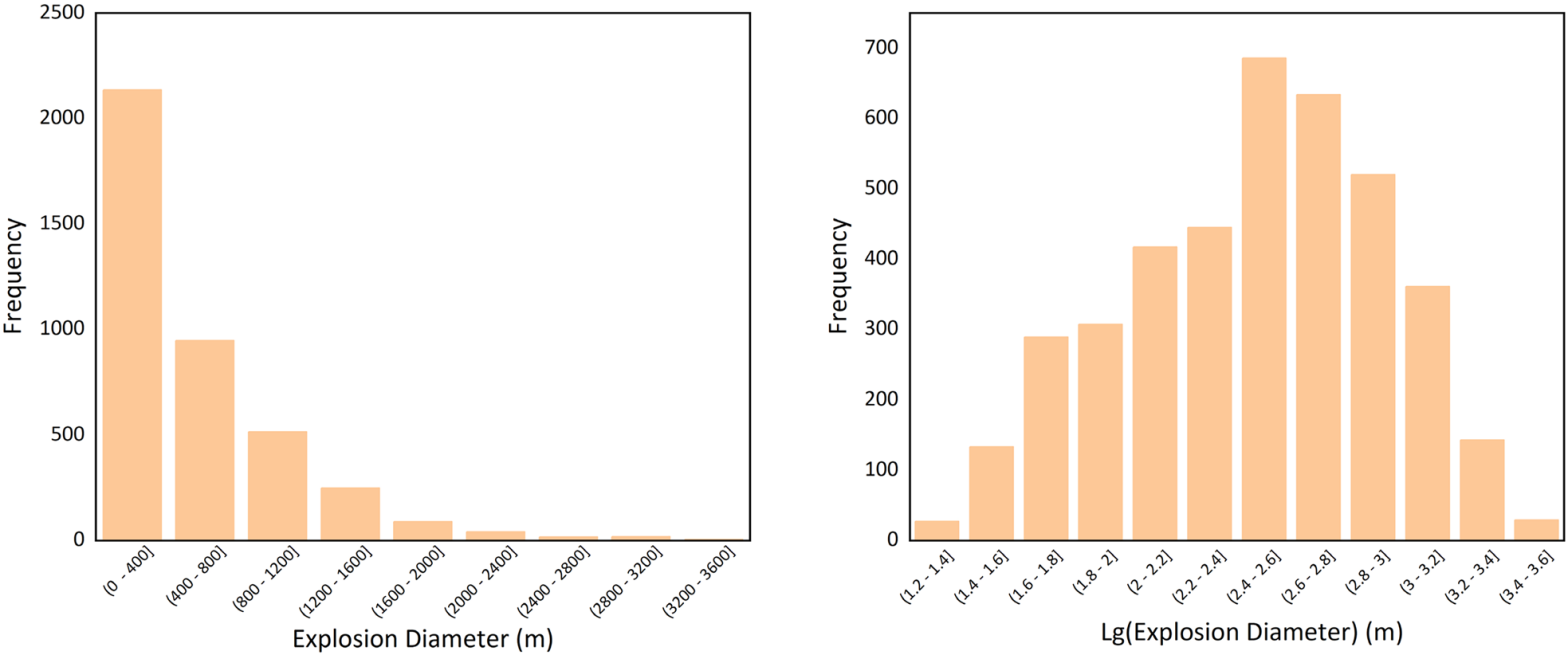
Histogram plot of explosion diameter model

Prior to developing the BTS model for explosive chemicals, it is essential to examine the chemical database to assess the feasibility of machine learning model development. This includes analyzing its distribution and statistical characteristics to determine the necessity of preliminary data transformation. As shown in Fig. 2, the database histogram reveals a highly skewed data distribution, which is highly detrimental to regression model development. Consequently, the data was processed using logarithmic transformation. The transformed data approximates a normal distribution, with its skewness significantly reduced, making it more suitable for machine learning and deep learning model development.

**Fig. 3:**
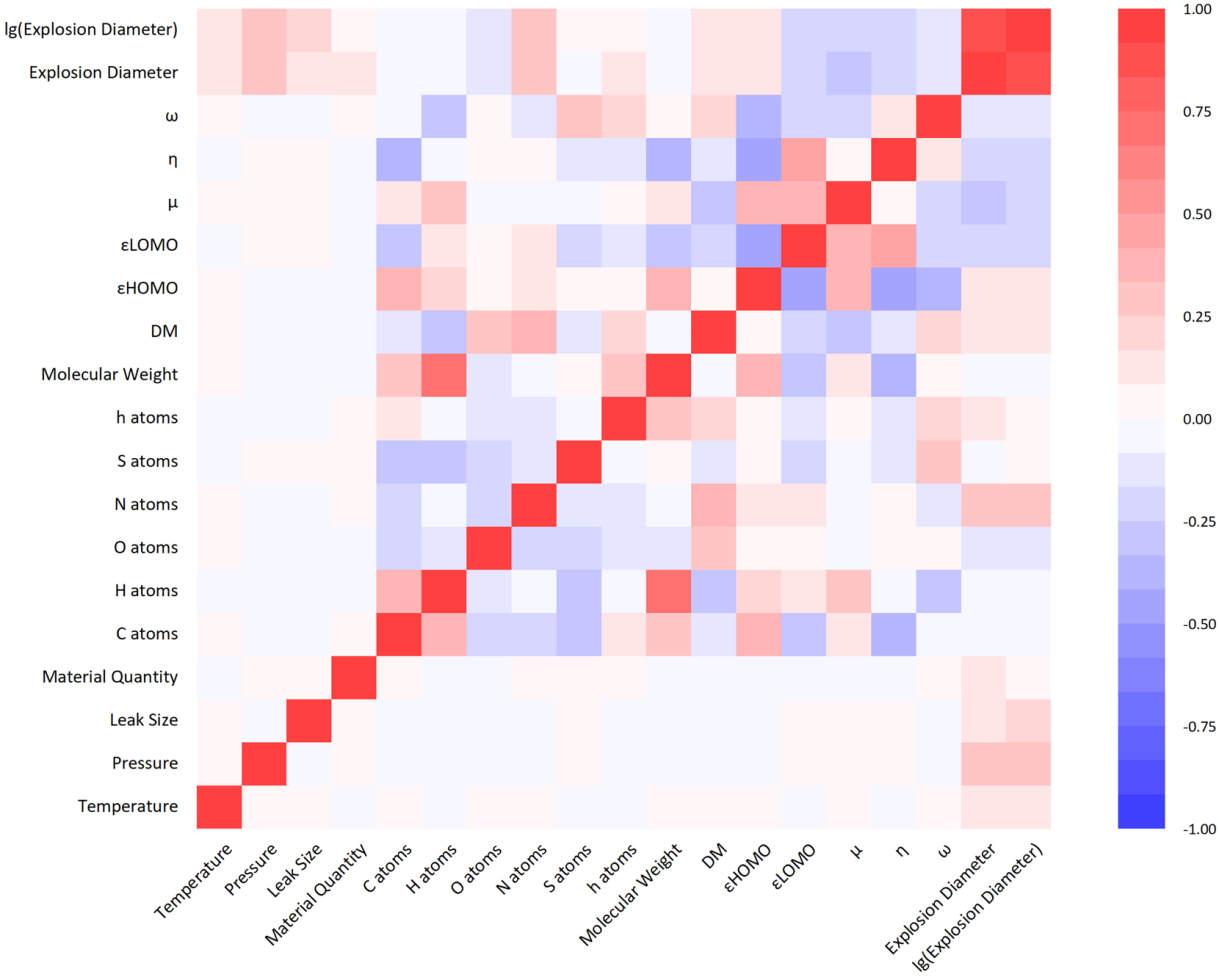
Correlation matrix of explosion model Variables

Furthermore, correlations among independent variables were examined to eliminate highly correlated features. Figure 3 shows the descriptor correlation matrices, revealing Pearson coefficients between −0.25 and 0.25, indicating no multicollinearity. Critically, preprocessing preserved the correlation between transformed descriptors and the target variable, confirming its effectiveness. The dataset was then randomly split into training and test sets using an 0.8:0.2 ratio, yielding 32 and 8 compounds respectively. The training set is used for model tuning and training, while the test set is reserved exclusively for a single final evaluation after hyperparameter and architecture optimization, ensuring objective assessment of the model’s actual prediction accuracy.

### Bayes-Transformer-SVM model performance evaluation

After completing the data transformation and the correlation analysis of input variables, a rigorous comparative analysis was conducted between the benchmark architecture and the novel Bayes-Transformer-SVM hybrid framework. As delineated in Table 2, the predictive efficacy of explosion diameter estimation was systematically characterized through multiple evaluation metrics. The Bayes-Transformer-SVM integration achieved superior coefficient of determination (R^2^ = 0.9475) coupled with minimal root mean square error (RMSE = 0.1139), quantitatively verifying its enhanced predictive fidelity over conventional approaches. Comparative computational analysis revealed that the Bayes-Transformer-SVM architecture exhibits statistically significant superiority in regression tasks when contrasted with baseline models, particularly in capturing nonlinear relationships within high-dimensional feature spaces.

**Table 2:** Statistical evaluation indicators of the predictive model

Input variables	Molecular descriptors			SMILES			Molecular descriptors & SMLIES
Metric	SVM	BPNN	Bayes-Transformer-SVM	SVM	BPNN	Bayes-Transformer-SVM	Bayes-Transformer-SVM
*R*^*2*^	0.9062	0.8149	0.9279	0.9236	0.9081	0.9367	0.9475
${\text{Q}}_{\text{CV}}^{2}$	0.9047	0.8105	0.9238	0.9186	0.9024	0.9315	0.9431
*MSE*	0.0222	0.0439	0.0175	0.0181	0.0218	0.0152	0.0130
*MAE*	0.1061	0.1556	0.0784	0.0819	0.1099	0.0741	0.0690
*RMSE*	0.1491	0.2094	0.1316	0.1345	0.1475	0.1234	0.1139

For the prediction of explosion diameter, significant performance differences emerged among the baseline models. Among them, the one using SMILES coding shows better predictive ability than the model based on MD. It is notable that the MS-SVM model achieved the lowest MAE and RMSE scores, which were 0.0819 and 0.1345 respectively. These results indicate the inherent instability in the baseline model. On the contrary, the proposed BTS demonstrated consistent performance in all evaluation metrics, which was attributed to the synergistic combination of topological descriptors and Smile-derived molecular fingerprints in the feature space. This multimodal integration strategy enhances the robustness of prediction through attention-based feature recalibration and effectively alleviates the information loss that is prevalent in the single-modal framework.

**Fig. 4:**
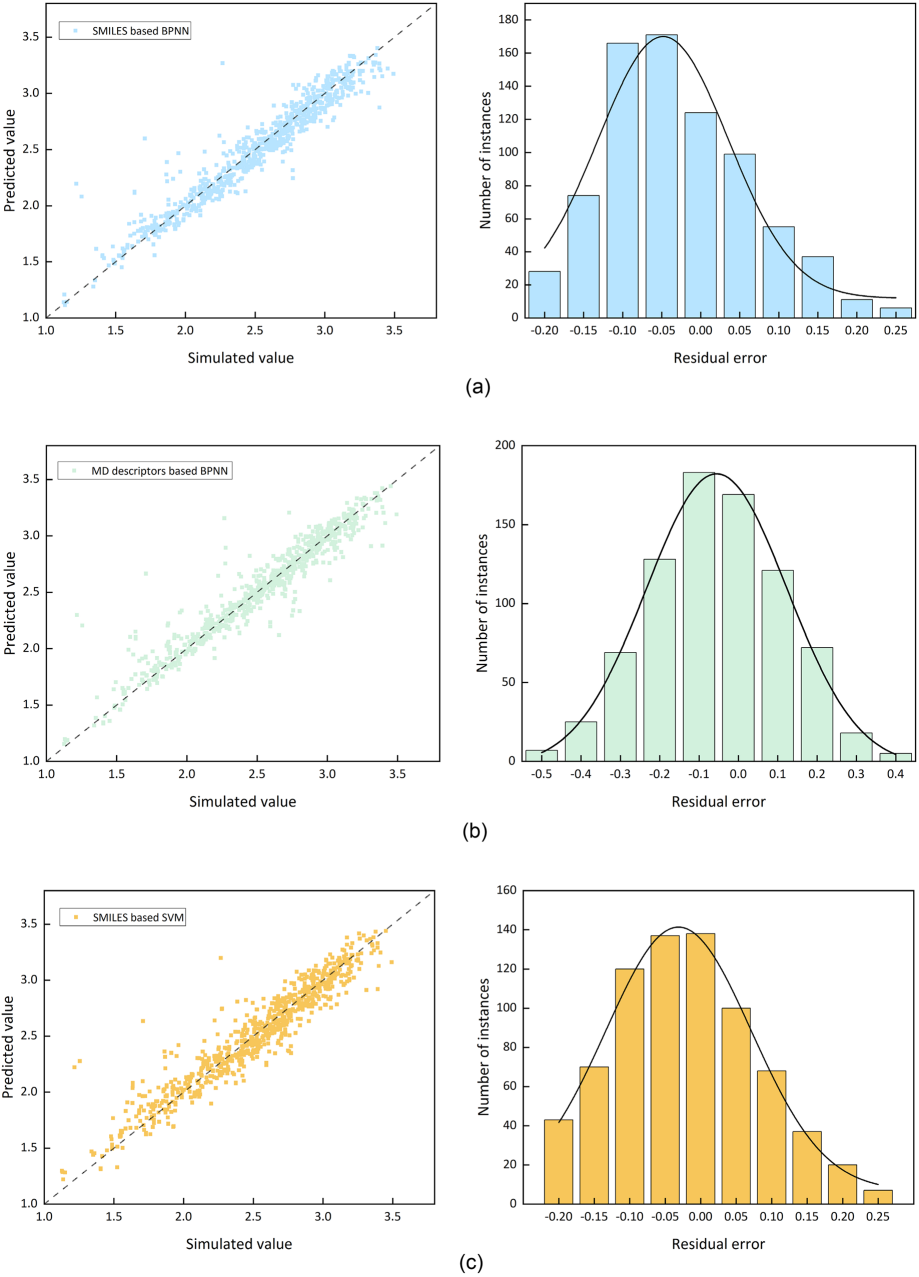

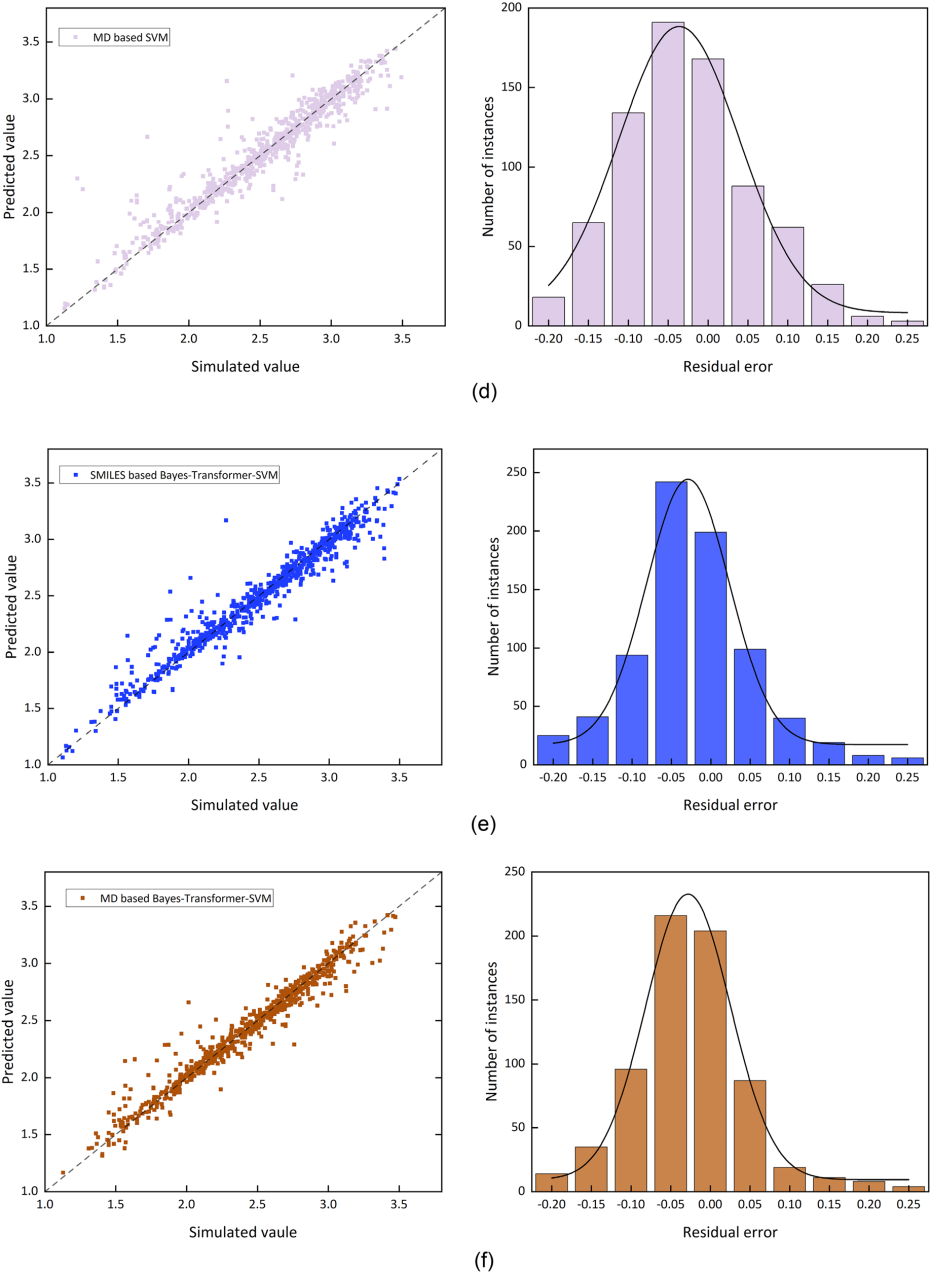

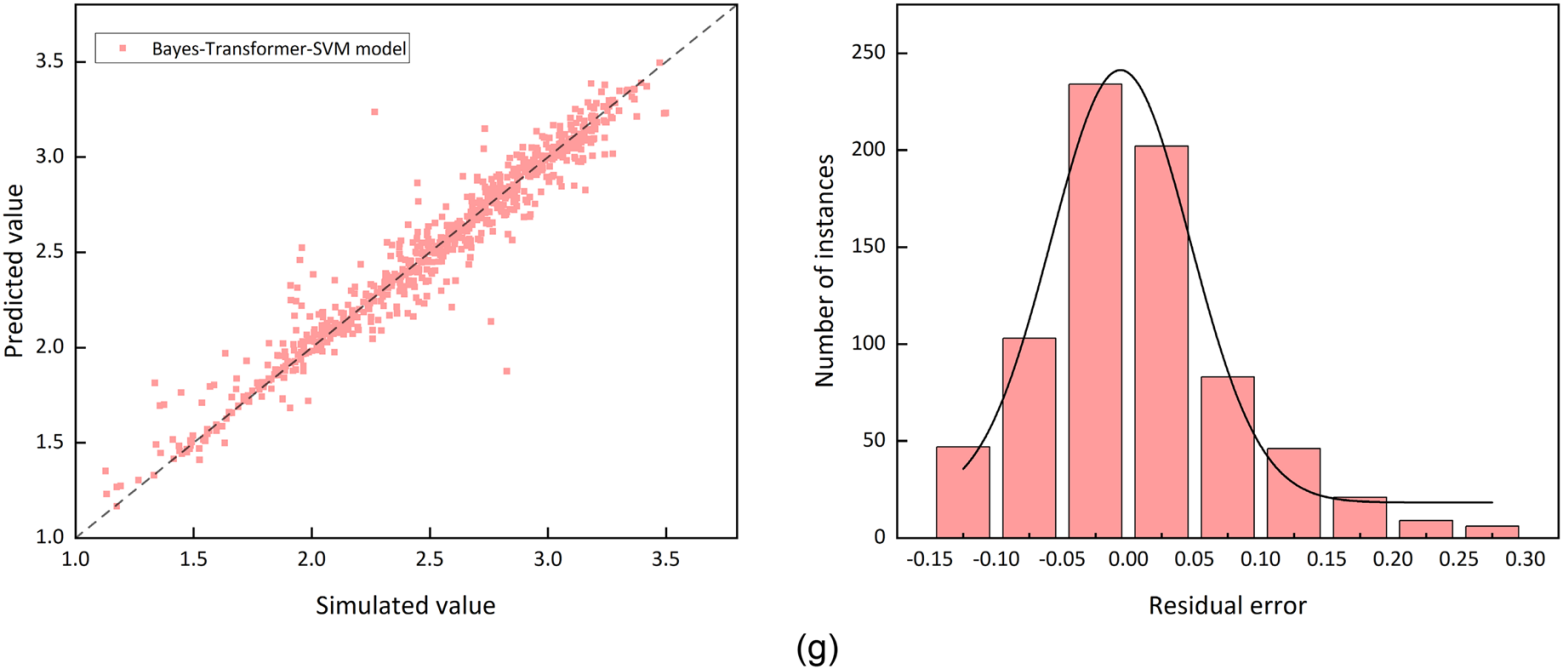
Model performance and residual histogram for different models

Figure 4 provides a visual assessment comparing the performance of the baseline model and the proposed Bayes-Transformer-SVM model. The figures show a logarithmic plot of the simulated data against the test set predictions, along with residual error plot.

Figure 4 demonstrates the close agreement between calculated and estimated values in the molecular descriptors-derived computational framework. The support vector machine algorithm exhibits superior predictive accuracy within the SMILES-based quantitative structure–activity relationship approach. When assessing the molecular descriptors descriptor model’s predictions, the data points exhibit greater dispersion across the regression plot, suggesting that atomic trajectory analysis proves inferior to structural descriptors in molecular representation efficacy. Notably, the hybrid methodology integrating molecular descriptors with chemical notation features in the Bayesian-optimized Transformer-SVM architecture displays minimal divergence from computational chemistry reference values, demonstrating enhanced predictive performance compared to single-descriptor models. 

Further performance analysis shows that the residuals of the SMILES-based model are mostly clustered between −0.05 and 0.05, which are more accurately predicted. In contrast, the residual distribution of the Bayes-Transformer-SVM model is tightly clustered around the zero-marker line, indicating a higher percentage of smaller errors. Therefore, the Bayes-Transformer-SVM model utilizing molecular descriptors and SMILES coding becomes the optimal model for predicting the explosion diameter. 

The selected Bayes-Transformer-SVM model refers to the validation criteria of the QSPR model, and ${\text{R}}_{0}^{2}$ and ${\text{R}}_{0}^{^{\prime}2}$ are used for evaluation. The values of relevant validation parameters of each model are shown in Table 3.

**Table 3:** Bayes-Transformer-SVM model performance verification

Metric	Molecular descriptors	SMILES	Molecular descriptors & SMILES
*R*^*2*^	0.9279	0.9367	0.9475
*R*_*0*_^*2*^	0.9264	0.9346	0.9465
*R'*_*0*_^*2*^	0.9243	0.9327	0.9447
*(R*^*2*^*-R*_*0*_^*2*^*)/R*^*2*^	0.0016	0.0022	0.0010
*(R*^*2*^*-R'*_*0*_^*2*^*)/R*^*2*^	0.0038	0.0042	0.0029
*k*	0.9245	0.9135	0.9424
*k'*	0.9386	0.9374	0.9787
*|R*_*0*_^*2*^*-R'*_*0*_^*2*^*|*	0.0021	0.0019	0.0018

The results of the study show that the metrics of the selected Bayes-Transformer-SVM model meet the validation criteria and can be used to predict the consequences of explosive accidents. The combination of molecular descriptors with SMILES for prediction has higher accuracy and is more suitable for prediction.

### Applicability domain by model

**Fig. 5:**
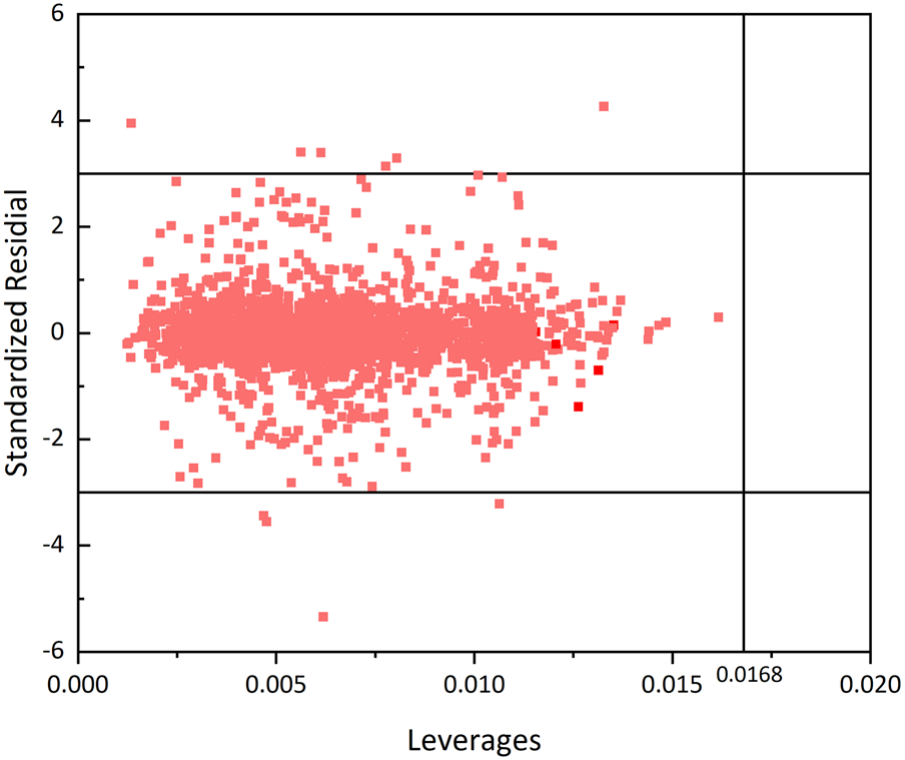
Applicability domain analysis results of Bayes-Transformer-SVM model (h* = 0.0168)

This section presents the AD of the proposed model by calculating the leverage values for each sample. Figure 5 represents the Williams plot of the leverage values, in which most of the data points fall within the applicable range of the model, and only a few of the data fall outside the range of ± 3 standard residuals, but none of them fall to the right of the standard leverage value h *, indicating that the model has a wide range of applicability and a high degree of reliability, and that it can be applied to the consequences of most of the hazardous chemicals explosion prediction of the consequences of the explosion of most hazardous chemicals.

### Ablation experiments

Ablation experiments evaluated the contribution of each module to the predicted performance by systematically removing key components of the model, as shown in Table 4. The full model (R^2^ = 0.9487, RMSE = 0.1126) demonstrated excellent predictive power, while a comparative analysis of different variants revealed the following key findings:

**Table 4:** Ablation experiments study results

Model variant	R^2^	ΔR^2^ vs full	RMSE
Full model	0.9487	–	0.1126
No cross-attention	0.8936	−5.51%	0.1621
No transformer	0.7577	−19.10%	0.2446

Removing the cross-attention mechanism results in a significant performance degradation (ΔR^2^ = −5.51%, RMSE = 0.1621), indicating its importance in capturing complex feature interactions. This suggests that cross attention plays a key role in modeling dependencies between different feature representations. The lack of Transformer architecture also contributes to performance degradation (ΔR^2^ = −19.10%, RMSE = 0.2446), which means that Transformer helps to improve the predictive power of the model, but may be more useful for local feature optimization than global modeling.

Taken together, model performance relies on the high order feature interaction capabilities of the cross-attention mechanism and the Transformer architecture provides robustness improvements.

### Mechanistic explanation of the model

The SHAP framework was employed to systematically evaluate the decision-making mechanism of the Bayesian-Transformer-SVM ensemble model, and the importance of each feature in the model is quantified by SHAP method.

**Fig. 6:**
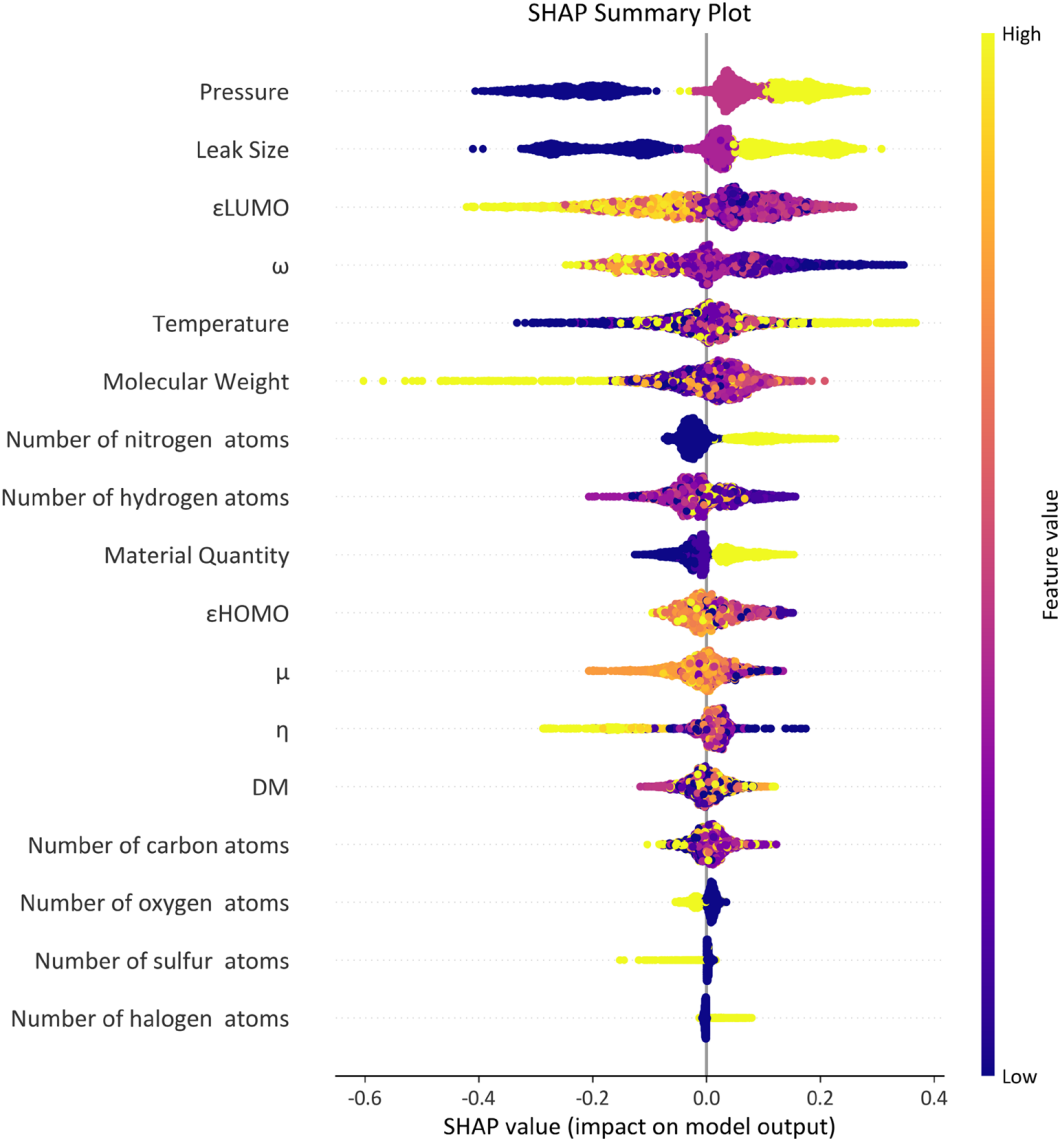
SHAP summary plot of input features

In this section, the SHAP method is adopted to analyze the feature contribution law of the prediction model. As shown in Fig. 6, Pressure is the core positive driving factor, among which the SHAP value of the pressure parameter (0.3 ± 0.04) quantifies its nonlinear effect. To explain the mechanism, it was further explored whether the identified key characteristic trends were consistent with the known physicochemical and engineering principles of the consequences of chemical explosions. The positive contribution of Material Quantity conforms to the basic physical principle that more combustibles contain greater energy and lead to more serious consequences. The positive impact of higher pressure is also consistent with engineering knowledge, as it increases the leakage rate and affects the formation/energy of vapor clouds. An increase in the ε_HOMO_ (maximum occupied molecular orbital energy) value (indicating that the molecule is more prone to losing electrons) is positively correlated with a larger explosion diameter, which supports the view that the inherent instability of the molecule may lead to more serious consequences under simulated conditions and is in line with the concept of reactivity.

## Conclusions

This paper presents a novel interpretable multimodal feature fusion BST (Bayes-Transformer-SVM) model for predicting compound explosion consequences. The key innovation lies in its unique integration of molecular-level descriptors (key molecular descriptors and SMILES codes) with operational parameters (leakage scenarios) as multimodal input. Leveraging an advanced multi-encoder architecture, the model not only achieves high predictive accuracy but also identifies and interprets critical decision features influencing the outcome. Methodologically, explosion scenarios under diverse leakage conditions were simulated using PHAST software to establish a comprehensive consequence database. Significantly, benchmarking against traditional SVM and BPNN models revealed their limited capability in linking simulation outputs to empirical observations, underscoring the need for our advanced approach.

Hyperparameter optimization within the BST framework yielded exceptional predictive performance (R^2^ = 0.9475, RMSE = 0.1139, MAE = 0.0690), demonstrating a robust consistency between computational predictions and experimental data. This work provides a pioneering computational toolkit for molecular-level explosion consequence prediction, reducing reliance on costly large-scale testing. It offers considerable potential for simulating active explosions and formulating preventive strategies, thereby strengthening industrial safety protocols and mitigating catastrophic risks.

## Supplementary Information

Supplementary material 1.

Supplementary material 2.

## Data Availability

No datasets were generated or analysed during the current study.
