# Supplementary material for: Prediction model for chemical explosion consequences via multimodal feature fusion
Source: J Cheminform. 2025 Aug 5;17:118. doi: 10.1186/s13321-025-01060-x (PMC12323191; doi:10.1186/s13321-025-01060-x)
Supplement: Supplementary file 1 — Supplementary material 1. [file 13321_2025_1060_MOESM1_ESM.docx]

Predicting Chemical Explosion Accidents based on Quantitative Structure-Consequence Relationship Analysis

***Supporting information***

Yilin Wang^1^, Beibei Wang^1, *^, Yichen Zhang^1^, Jiquan Zhang^2^, Yijie Song^1^, Shuang-Hua Yang^3^

^1^ College of Jilin Emergency Management, Changchun Institute of Technology, Changchun 130012, China.

^2^ School of Environment, Northeast Normal University, Changchun 130117, China.

^3^ Department of Computer Science, University of Reading, Reading RG6 6AH, UK.

Corresponding author:

Name: Beibei Wang

E-mail: wangbeibei@ccit.edu.cn

**Table S1** Symbols of molecular descriptors in this study.

| notation | define |  |
| --- | --- | --- |
| MW | Molecular Weight, g/mol |  |
| n_C_ | Number of Carbon Atoms |  |
| n_H_ | Number of Hydrogen Atoms |  |
| n_O_ | Number of Oxygen Atoms |  |
| n_N_ | Number of Nitrogen Atoms |  |
| n_S_ | Number of Sulfur Atoms |  |
| SP | Structural Parameters |  |
| DM | Dipole Moment, D |  |
| ε_HOMO_ | The Energy of The Highest Occupied Molecular Orbital, a. u. |  |
| ε_LUMO_ | Minimum Occupied Molecular Orbital Energy, a. u. |  |
| μ | Chemical Potential, a. u. | $\mu=\frac{(\varepsilon_{HOMO}+\varepsilon_{LUMO})}{2}$ |
| η | Hardness, a. u. | $\eta=\varepsilon_{LUMO}-\varepsilon_{HOMO}$ |
| ω | electrophilic index, a. u. | $\omega=\frac{\mu^{2}}{2\eta}$ |


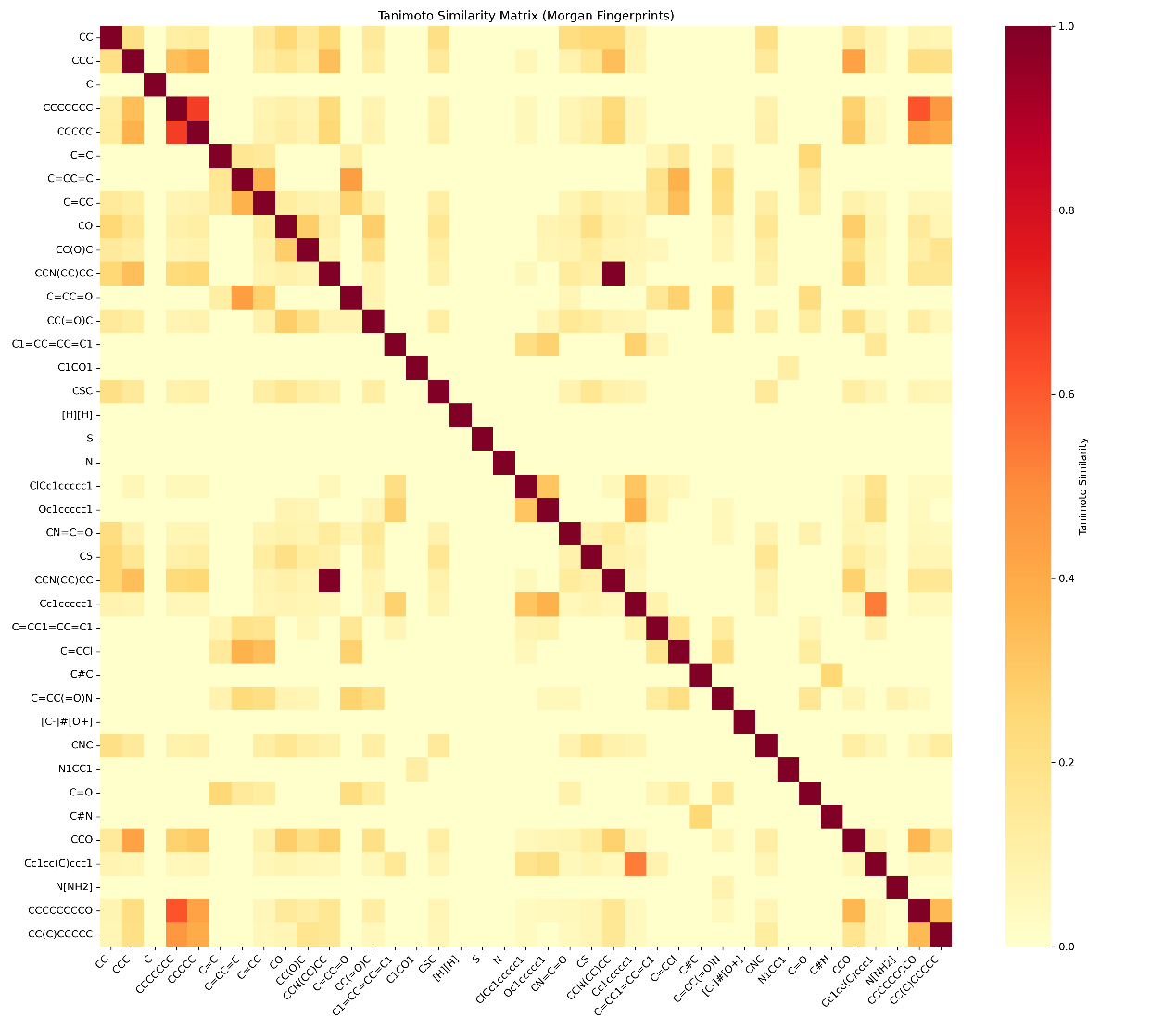


Figure.S1 Tanimoto similarity matrix plot
